# Use of biogenic silver nanoparticles on the cathode to improve bioelectricity production in microbial fuel cells

**DOI:** 10.3389/fchem.2023.1273161

**Published:** 2023-09-22

**Authors:** Ismail Elkhrachy, Vandana Singh, Ankit Kumar, Arpita Roy, Mohamed Abbas, Amel Gacem, Mir Waqas Alam, Krishna Kumar Yadav, Devvret Verma, Byong-Hun Jeon, Hyun-Kyung Park

**Affiliations:** ^1^ Civil Engineering Department, College of Engineering, Najran University, Najran, Saudi Arabia; ^2^ Department of Microbiology, SSAHS, Sharda University, Greater Noida, Uttar Pradesh, India; ^3^ Department of Life Sciences, School of Basic Sciences and Research, Sharda University, Greater Noida, India; ^4^ Department of Biotechnology, Sharda School of Engineering and Technology, Sharda University, Greater Noida, India; ^5^ Electrical Engineering Department, College of Engineering, King Khalid University, Abha, Saudi Arabia; ^6^ Department of Physics, Faculty of Sciences, University 20 Août 1955, Skikda, Algeria; ^7^ Department of Physics, College of Science, King Faisal University, Al-Ahsa, Saudi Arabia; ^8^ Faculty of Science and Technology, Madhyanchal Professional University, Bhopal, India; ^9^ Environmental and Atmospheric Sciences Research Group, Scientific Research Center, Al-Ayen University, Nasiriyah, Iraq; ^10^ Department of Biotechnology, Graphic Era Deemed to be University, Dehradun, Uttarakhand, India; ^11^ Department of Earth Resources and Environmental Engineering, Hanyang University, Seoul, Republic of Korea; ^12^ Department of Pediatrics, Hanyang University College of Medicine, Seoul, Republic of Korea

**Keywords:** oxygen reduction reaction, cathode modifier, biogenic, nanoparticles, microbial fuel cell

## Abstract

To date, research on microbial fuel cells (MFCs) has. focused on the production of cost-effective, high-performance electrodes and catalysts. The present study focuses on the synthesis of silver nanoparticles (AgNPs) by *Pseudomonas* sp. and evaluates their role as an oxygen reduction reaction (ORR) catalyst in an MFC. Biogenic AgNPs were synthesized from *Pseudomonas aeruginosa* via facile hydrothermal synthesis. The physiochemical characterization of the biogenic AgNPs was conducted via scanning electron microscopy (SEM), X-ray diffraction (XRD), and UV-visible spectrum analysis. SEM micrographs showed a spherical cluster of AgNPs of 20–100 nm in size. The oxygen reduction reaction (ORR) ability of the biogenic AgNPs was studied using cyclic voltammetry (CV). The oxygen reduction peaks were observed at 0.43 V, 0.42 V, 0.410 V, and 0.39 V. Different concentrations of biogenic AgNPs (0.25–1.0 mg/cm^2^) were used as ORR catalysts at the cathode in the MFC. A steady increase in the power production was observed with increasing concentrations of biogenic AgNPs. Biogenic AgNPs loaded with 1.0 mg/cm^2^ exhibited the highest power density (PD_max_) of 4.70 W/m^3^, which was approximately 26.30% higher than the PD_max_ of the sample loaded with 0.25 mg/cm^2^. The highest COD removal and Coulombic efficiency (CE) were also observed in biogenic AgNPs loaded with 1.0 mg/cm^2^ (83.8% and 11.7%, respectively). However, the opposite trend was observed in the internal resistance of the MFC. The lowest internal resistance was observed in a 1.0 mg/cm^2^ loading (87 Ω), which is attributed to the high oxygen reduction kinetics at the surface of the cathode by the biogenic AgNPs. The results of this study conclude that biogenic AgNPs are a cost-effective, high-performance ORR catalyst in MFCs.

## 1 Introduction

Microbial fuel cells (MFCs) are currently one of the most promising alternative methods of obtaining electricity through wastewater treatment (Shamsudin et al., 2022). Over the past 10 years, MFCs have attracted the attention of academia. In 2016, more than 75% of all studies published in the field of bioelectrochemical systems (BESs) were about MFCs. MFCs are a type of BES that purify wastewater while converting the chemical energy in the organic substrate into direct electrical energy (Hamouda et al., 2021). Direct electrical power is produced in an MFC through the simultaneous oxidization of biodegradable materials using exoelectrogens as biocatalysts and the reduction of oxygen at the anode and cathode electrodes, respectively. Because the cells in a stacked MFC reverse their voltages, the total voltage created by the stack is equal to or less than the total voltage generated by a single cell, which means that MFCs cannot be simply connected in series to raise the overall potential (Gnanamangai et al., 2022). MFCs convert organic waste into bioelectricity through the energization of microorganisms that act as a biocatalyst in this system (Mahmoud et al., 2022). MFCs are strongly dependent on several components to produce electricity (Rousseau et al., 2021). The type of electrode utilized and the make-up of the electron acceptors on the cathode are the main determinants of power generation. Power production depends on the reduction kinetics of the cathode. In aerobic MFCs, electron efficiency refers to how effectively electrons generated during microbial oxidation of organic matter at the anode are transferred and utilized for the reduction of oxygen at the cathode to produce electrical current. Oxygen is abundant in the environment and has a high redox potential. Dye, organic pollutants, and landfill leachate can be treated in a single chamber, double chamber, and tubular MFC using a variety of electrodes, including carbon, stainless steel, and graphite sheets. A single chamber MFC separated by a proton-selective Nafion membrane was used for the treatment of sulfate and organic-rich wastewater as well as the production of bioelectricity (Ranjan et al., 2022). In their exploration of the kinetics of pollutant removal, Elmaadawy et al. (2020) found that the electrode served as a carrier surface for the microbial population as well as an electron donor or acceptor and that it has a major impact on the efficiency of MFCs and the generation of bioelectricity. A slow decrease in the kinetics of oxygen reduction on the cathode has a negative effect on MFC performance (Liu et al., 2022). This problem is solved by using a variety of expensive and innovative metals and alloys (Pt, Ag, Au, and Pd) as cathode catalysts to accelerate the kinetics of the oxygen reduction reaction (ORR) (Wang et al., 2019). One of the most significant chemical processes occurs at the cathode of a fuel cell and has a synergistic effect on overall performance. Due to their effectiveness and high electrocatalytic activity, platinum (Pt) and Pt-based materials have recently been used as ORR catalysts on the MFC surface of cathodes (Wang et al., 2021). However, due to its high price, Pt is not suitable for commercial MFC applications. As a result, it serves as a catalyst in the creation of additional low-cost electrocatalysts, including graphene that has been functionalized with iron and nitrogen, manganese (IV), and magnesium oxide, which is supported by graphene oxide (Dong et al., 2021). As a result of the high cost and high toxicity, catalyst use has been constrained; as a result, it has become a barrier to commercialization. To overcome this limitation, several catalysts for increasing ORR on the cathode—which also inhibits certain nanoparticles —have been developed in recent years. There is still a need for a bifunctional cathode catalyst that will inhibit the growth of unwanted microbes, thereby enhancing ORR (Morris et al., 2007; Hassan et al., 2022).

Nanoparticles are highly stable, inexpensive, and have good redox properties, which are important for the ORR on MFC cathodes. For better ORR, many transition metals and their oxides are being investigated. Recently, silver-based compounds have been investigated as potential ORR catalysts. However, there is little research on biogenic AgNPs used as ORR catalysts. Biogenic silver has a low conductivity, and there are not many surface-active locations available for this use. Therefore, it is necessary to create biogenic silver with a structure that can increase ORR electrocatalytic activity. The ORR catalytic efficiency might be significantly increased by increasing both the number of active sites and the inherently reactive nature of those sites. The electrocatalytic activity of these ORR-active sites can be increased by contacting the catalyst surface with an oxygen-deficient chemical. The addition of an oxygen-deficient molecule may significantly alter the surface’s electrical characteristics and reduce the absorption energy of H_2_O, which is crucial to the ORR’s catalytic activities. As a result, an oxygen-deficient catalyst would increase the number of active sites, provide catalytic activity, and enhance ORR activity. The relationship between active sites and ORR activity is made clearer due to this research.

The antibacterial properties of AgNPs are intricate and involve numerous mechanisms (Mubara et al., 2011; Nagajyothi and Lee, 2011; Huy et al., 2013; El-Sheekh et al., 2022). It is still unclear how the antibacterial action works. Nanoparticles have been shown to have antimicrobial effects at low doses. The literature has identified several likely mechanisms (Sahayaraj and Sathiyamoorthy, 2011) that lead to cell death, including 1) oxidative stress through reactive O_2_ species (ROS) generation, 2) dissolution and ion release, and 3) internalization of these nanometals in nuclei (Ahmed et al., 2016). The main mechanism involves the production of ROS, which is related to the particle size, surface area, and crystallinity of the NPs. AgNPs typically interact negatively with bacteria, and this interaction has been exploited for antimicrobial uses in industries including food and agriculture. Using bactericidal AgNPs as a replacement for some standard antibiotics may help address the developing issues of antibiotic resistant strains produced by the spread of antibiotic resistance genes among bacteria (Sahayaraj and Sathiyamoorthy, 2011; Ahmed et al., 2016).

In this work, the ORR catalytic performance of biogenic AgNPs on the cathode surface was investigated. The physiochemical characterization of biogenic AgNPs was conducted. Different concentrations of AgNPs (ranging from 0.25 to 1.0 mg/cm^2^) were used as an ORR catalyst on the cathode, and their electrochemical performance was investigated in an MFC.

## 2 Materials and methods

### 2.1 Biogenic silver preparation

*Isolation of bacteria strain:* A 1.0 g aliquot of a soil sample was taken with 10 mL of sterile deionized water. The soil solution was serially diluted to 10^−5^. An 80 μL aliquot of the 10^−5^ diluted solution was inoculated and incubated for 24 h at 37°C. Following incubation, colonies with distinct morphologies were recovered, isolated, and identified using procedures from the *Bergey Manual of Determinative Bacteriology* and further identified using 16S RNA technology (Figure 2). Following isolation, a pure culture of *Pseudomonas sp*. was kept and stored at 4°C for further use.

*Chemical preparation:* A 5 mM solution of AgNO_3_ (molar mass = 170) was prepared using 0.84 g of AgNO_3_ and 1000 mL of deionized water.

**FIGURE 1:**
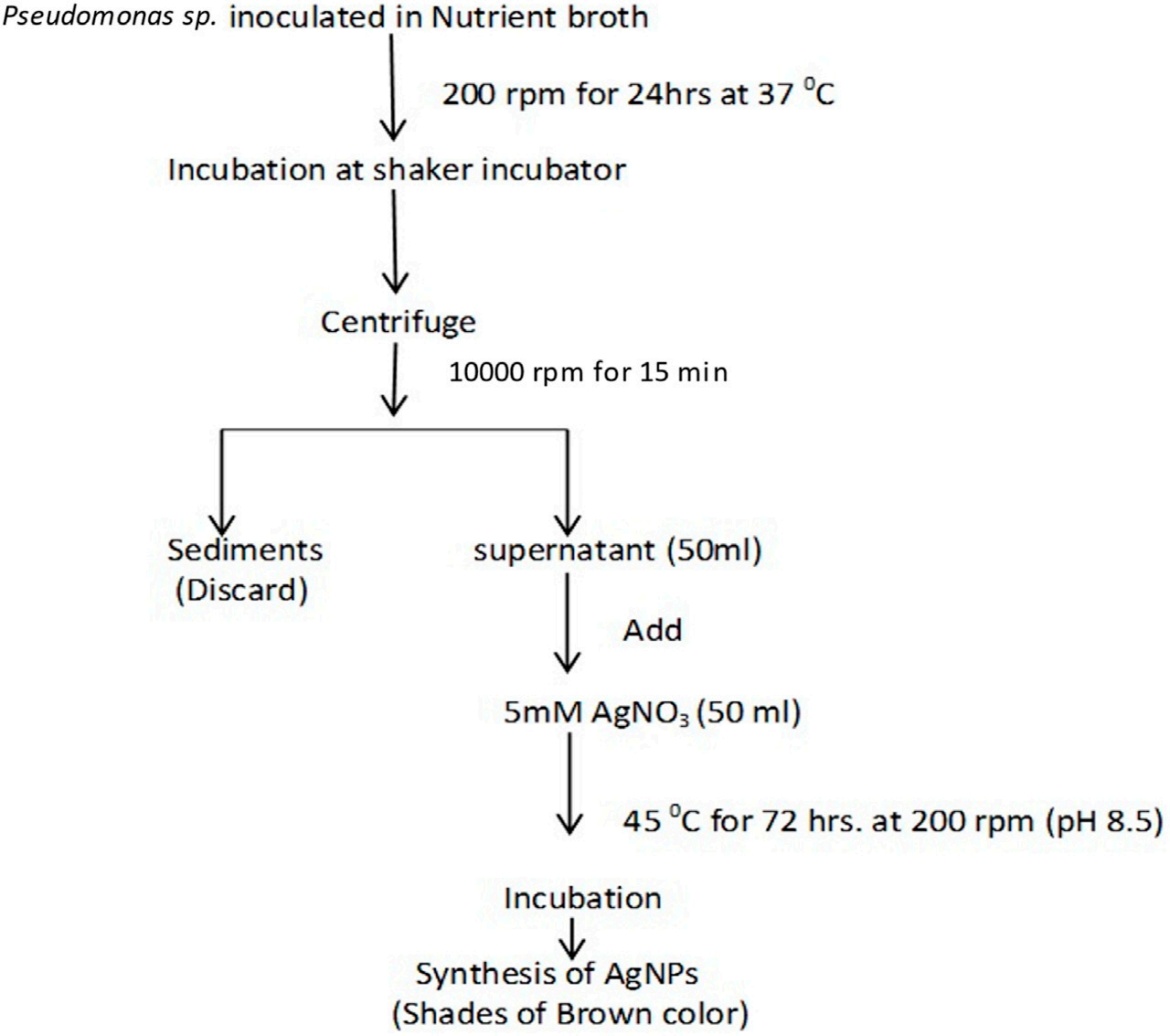
Basic procedure (flow chart) of the experiment for the formation of biogenic AgNPs.

*Procedure for synthesis:* AgNPs were synthesized using the facile hydrothermal synthesis method. *Pseudomonas aeruginosa* was employed to create the AgNPs used in this study. A loop full of bacterial culture was added to 1 mL of sterile normal saline solution to create the bacterial inoculum. The same inoculum was then inoculated in 250 mL of nutrient broth (NB) (HiMedia) and cultured in the BOD incubator for 24 h at 37°C. The culture was centrifuged at 10,000 rpm for 15 min at 25°C. The supernatant was collected and filtered using filter paper (Masum et al., 2019). The pH was adjusted to 8.5. A 50 mL aliquot of freshly prepared 5.0 mM AgNO_3_ was mixed with 50 mL of the supernatant, and the resulting mixture was incubated at 45°C for 72 h at 200 rpm. AgNP production is indicated by a change in color. The basic procedure for AgNP synthesis can be seen in Figure 1.

### 2.2 Physical characterization of biogenic AgNPs

Crystallinity and crystal structure were evaluated using X-ray diffraction (XRD) analysis with Cu Kα radiation. The surface morphology of the materials was examined using SEM. A solution containing 2% formaldehyde and 0.02% picric acid in 0.1 M sodium phosphate buffer, pH 7.2, was used to fix the cells to the electrode surface. The samples were dehydrated in an ethanol gradient of 40%–100% for 5 min each and then retained in desiccators for later use. To achieve a constant coating thickness of 300–350 Å, gold ions were sputtered in a Hitachi E101 ion sputterer operating at a vacuum of 0.1–0.01 Torr. The materials produced for this study were SEM-imaged using a ZEISS Supra 40 scanning electron microscope with an incident electron beam intensity of 10 keV and a working distance of 6 mm (Ibrahim et al., 2021).

### 2.3 Electrode preparation

The electrodes were prepared by incorporating different concentrations of biogenic AgNPs (0.25, 0.5, 0.75, and 1.0 mg/cm^2^) into the cathode following the aforementioned synthesis. Acetone/isopropanol (40 mL) was mixed with biogenic AgNPs. Nafion (10 L), a proton exchange separator, and PTFE solution (0.5 mL), a binder, were added to the mixture. The resulting solution was sonicated in a bath sonicator for an hour before being applied to carbon felt with a gravity spray cannon (Noida graphite 5 mm). The biogenic AgNPs were dried on carbon felt for 1 h at 80°C (Magar et al., 2021).

### 2.4 MFC construction

The MFC consisted of an anode compartment and a membrane cathode assembly (MCA) placed on opposite sides. The anode chamber comprised a cuboidal chamber made of transparent polyacrylic material with outer dimensions of 7 cm × 8 cm × 3.5 cm with a capacity of 110 mL. The anode chamber had two ports at the top, one for the electrode terminal and the other for the reference electrode (Ag/AgCl, saturated KCl; +197 mV, Equiptronics, India) and sampling. The anode consisted of a carbon cloth with a working surface area of 12 cm^2^ with a stainless steel wire welded to form the terminal. The membrane cathode assembly (MCA) was prepared by coating the membrane with a catalyst-loaded cathode. To prepare the cathode, conductive ink containing a cathode catalyst [0.15 mg/cm^2^ laboratory-prepared biogenic silver dust and carbon black VULCAN XC-72 (0.35 mg/cm^2^; Cabot Corp.; India)] was taken in a 20 mL 1:1 acetone-isopropyl alcohol solution with 0.3 mg/cm^2^ PVA (1% w/v) aqueous solution as a binder. Ultrasonication of the PVA–biogenic AgNP-loaded carbon black aqueous acetone solution was done for 30 min and used as ink to spread on the cathode. The ink containing the cathode catalyst was sprayed onto the preheated membrane, which was kept in the oven at 60°C. The MCA was manufactured by bonding the carbon ink-coated anion exchange membrane (AEM) [consisting of polyvinyl alcohol (PVA) and poly(diallyldimethylammonium) chloride (PDDA)] directly onto a flexible stainless steel (SS) mesh as a current collector. SS mesh (8 cm^2^) was attached to all the membranes using conducting paint (Siltech Corp., India) on the side facing the air. The SS mesh used in the present study was the SS-304 type with 50 × 50 openings per square inch (wire diameter 0.17 mm). It was connected to a concealed copper wire as a cathode terminal.

Concealed copper wires were used to connect the external resistance of 100 Ω to close the circuit. The inter-electrode distance was kept constant in all the experiments (∼2.5 cm); the anodes were placed equidistant from the MCA. The additional ports were sealed with clamped tubes to ensure an anaerobic environment. The MFCs were washed with 70% alcohol and kept in the UV chamber for 30 min before the experiment.

### 2.5 Electrochemical measurements of biogenic AgNP electrodes

Linear sweep voltammetry (LSV) is frequently employed in MFCs to examine the ORR activity of electroactive materials. The LSVs of the biogenic AgNP cathodes were recorded at a scan rate of 1 mV/S (Algebaly et al., 2020). Three electrodes—a biogenic AgNP cathode, a Pt wire, and Ag/AgCl—served as working, counter, and reference electrodes in the electrochemical experiments. The same electrode setup was used for electrochemical impedance spectroscopy (EIS) of a biogenic AgNP cathode.

### 2.6 Biofilm formation studies on biogenic AgNP cathodes

Biofilm formation on the biogenic AgNP cathode was studied for six batch cycles. The average biovolumes of dead cells, living cells, and extracellular polymeric substances (EPS) for each kind of sheet were calculated and plotted for evaluation using different concentrations of AgNPs (0.25 and 1.0 mg/cm^2^), without 0 mg/cm^2^ loading of AgNPs used as a control.

After the biofilm development experiment, the various anodes were carefully removed, and 5 mm × 5 mm samples were cut from a consistent location on each anode. A phosphate-buffered saline (PBS) solution containing 0.1 mg/mL, 3 mM propidium iodide (PI) was used as a staining solution. The biofilms were incubated in the staining solution for 30 min in the dark until they were stained. A confocal laser scanning microscope (CLSM; ZeissMeta510; Carl ZEISS, Inc., United States) equipped with a ZEISS dry objective LCI Plan-Neofluar was used to observe the stained biofilm samples (×20 magnification and a numerical aperture of 0.5). Images were taken at ten different places on each surface and were stitched together. The process previously described in Munoz-Cupa et al. (2021)was used for image capture and processing. COMSTAT, an image-processing program in MATLAB 6.5 (MathWorks, Inc., Natick, MA), was used to examine the photos and calculate the precise biovolume (m^3^/m^2^) in the biofouling layer (Kumar et al., 2023). Ten places on each biogenic AgNP electrode were chosen for microscopical observation and analysis. The CLSM image stacks were 3-dimensionally rebuilt using the Imaris program (Imaris Bitplane, Zurich, Switzerland). The average biovolumes of living, dead cells, and EPS were determined and analyzed for the different concentrations of the AgNP electrodes, including the control.

## 3 Result and discussion

### 3.1 Isolation and screening of *Pseudomonas aeruginosa*

**FIGURE 2:**
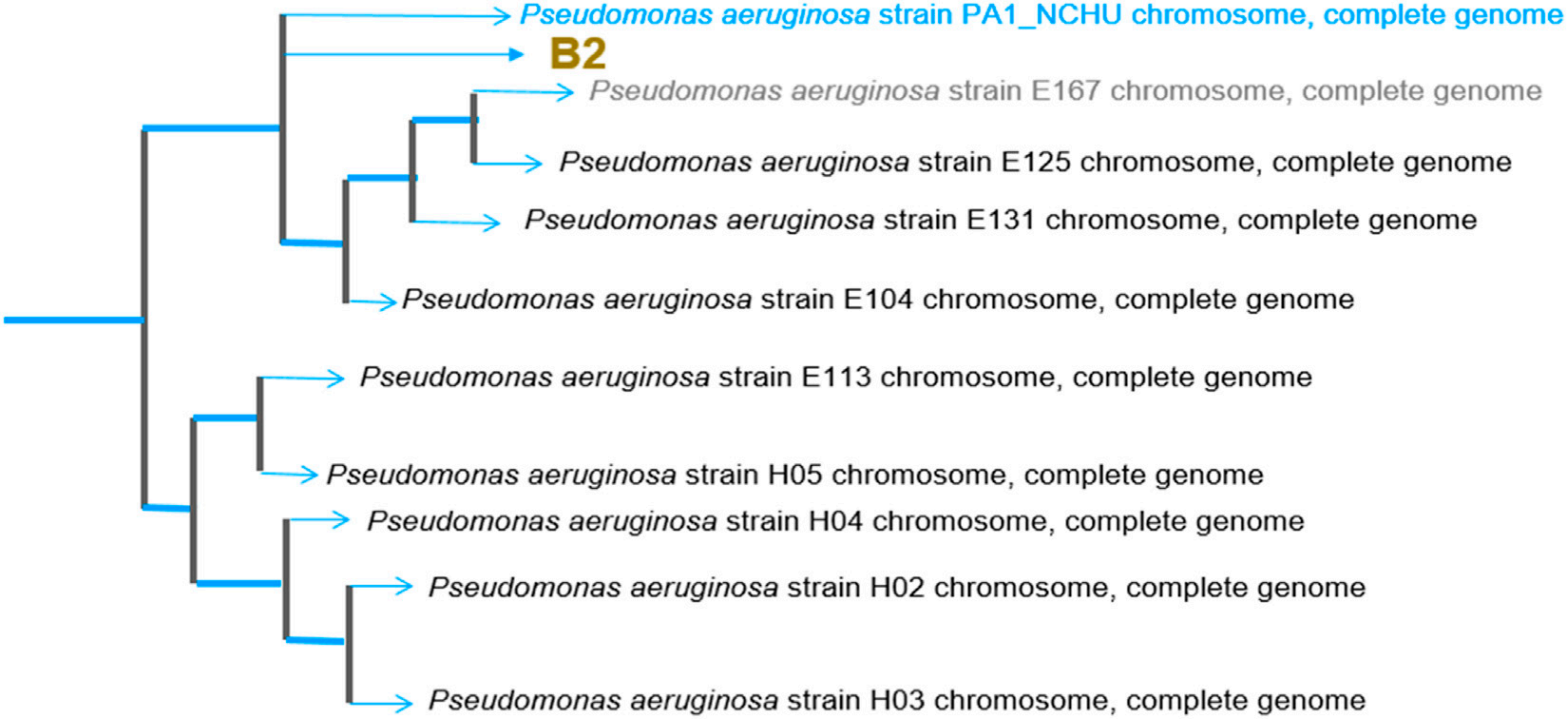
Phylogenetic tree of *Pseudomonas aeruginosa* isolates.

The culture isolates were identified using 16S rRNA analysis and confirmed as *Pseudomonas aeruginosa*. The Basic Local Alignment Search Tool (BLAST) program was used to find the species most closely similar in the database to the DNA sequences of the bacteria under study. For *P*. *aeruginosa*, the best match was with the species and the query sequence with accession number CP093395.1 (Figure 2).

### 3.2 Scanning electron microscopy

**FIGURE 3:**
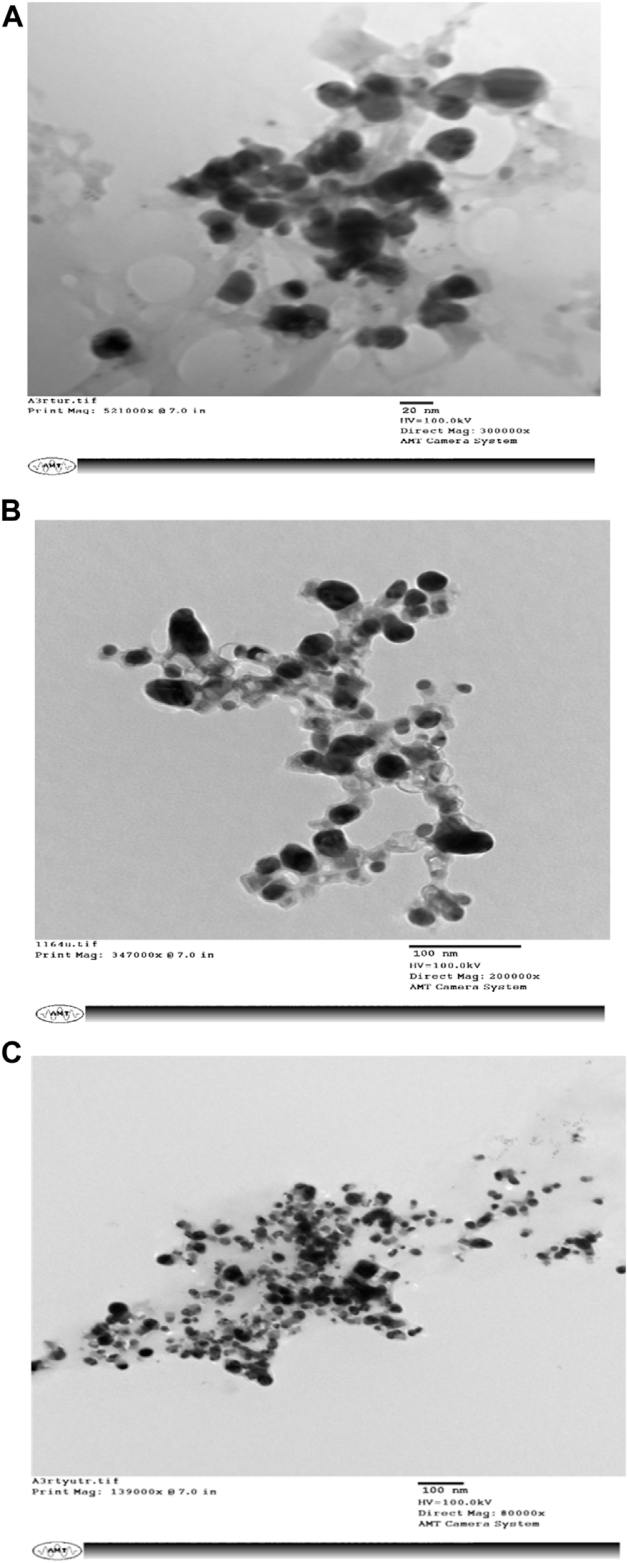
**(A–C)** depict Scanning Electron Microscopy (SEM) images of biologically synthesized silver nanoparticles (AgNPs) that exhibit a striking diversity in their sizes.

SEM micrographs shown in Figure 3 reveal that the biogenic AgNPs are 20–100-nm spherical clusters.

### 3.3 XRD of silver nanoparticles

**FIGURE 4:**
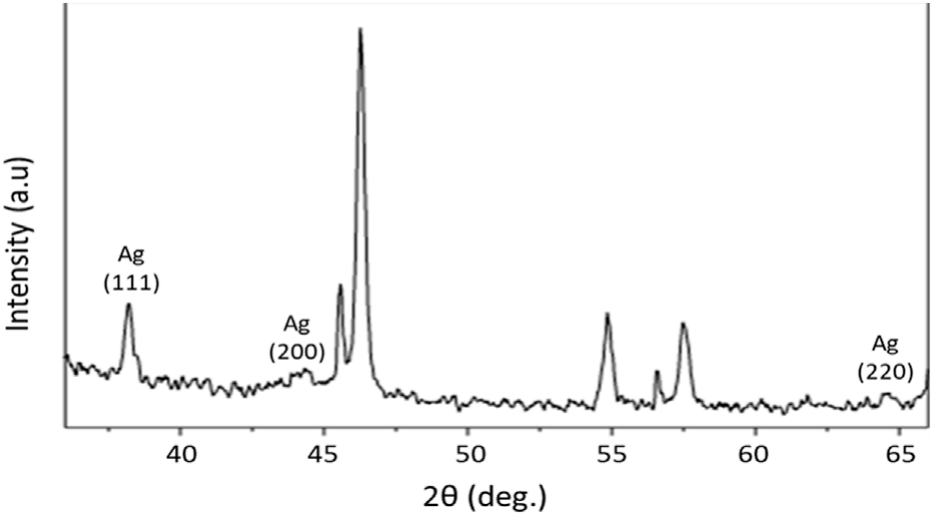
XRD analysis of AgNPs: the XRD pattern of AgNPs shows that all diffraction peaks resemble the characteristic FCC silver lines. The distinctive diffraction peak at approximately 38°, which shows the index 111, was a clear indication of cubic face-centered silver. Broader peaks signify smaller particle sizes.

The apparent peaks in the XRD patterns of AgNPs indicate the specific crystallographic planes present within the crystalline lattice of the nanoparticles. In the context of a cubic crystal system, such as the face-centered cubic (FCC) structure commonly observed in AgNPs, it is customary to designate peaks using indices such as (111), (200), and (220). The observed peak numbers in the XRD patterns of AgNPs indicate the crystallographic planes present within the lattice structure of the nanoparticles and their orientation with respect to the crystal axes. The numerical values, denoted as Miller indices, provide a crucial scientific understanding of the atomic configuration of nanoparticles and serve as the basis for determining their crystallographic structure and properties. The XRD analyses of biogenic AgNPs can be seen in Figure 4.

### 3.4 Production of AgNPs

**FIGURE 5:**
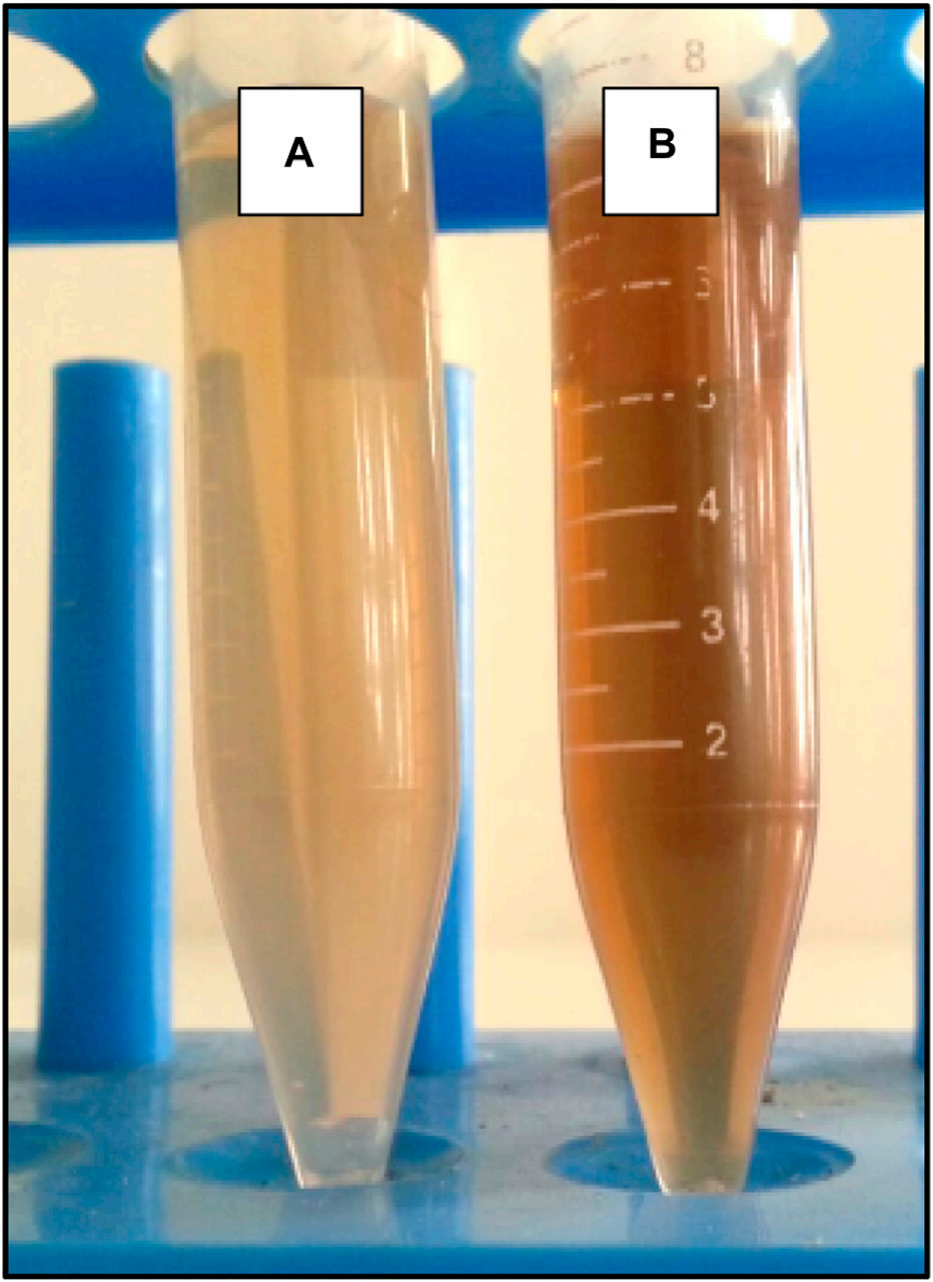
Synthesis of AgNPs: Tube **(A)** contains the supernatant obtained from a *Pseudomonas* bacterial culture. Tube **(B)** demonstrates observable indications (brown) of the synthesis of AgNPs.

The color change indicates the synthesis of AgNPs. Tube A represents the supernatant harvested from *Pseudomonas* sp., and tube B shows the formation of AgNPs (Figure 5).

### 3.5 UV–visible spectrum of AgNPs

**FIGURE 6:**
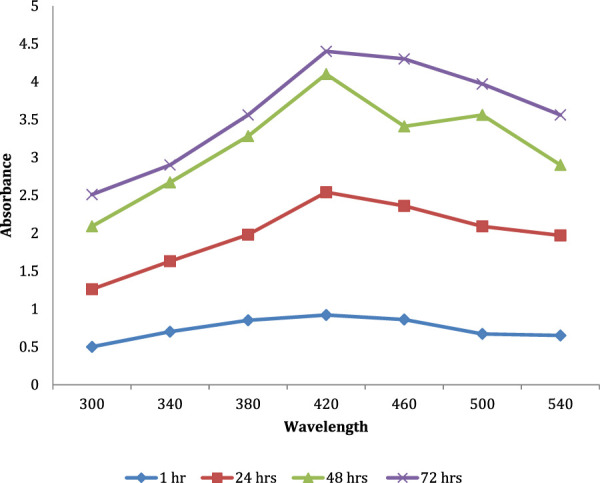
UV–vis analysis of the AgNPs. Biogenic AgNP UV–vis spectra were analyzed at 24 h, 48 h, and 72 h from 300 nm–540 nm. The color change from straw-yellow to reddish brown indicates the synthesis of AgNPs after 72 h at 45°C at 200 rpm in a shaker incubator. The spectra of AgNPs formed by surface plasmon vibrations had the greatest absorption at 420 nm.

UV–vis spectra analysis of biogenic AgNPs was performed, as seen in Figure 6, over a range of 300–540 nm at intervals of 24 h, 48 h, and 72 h. The color of the synthesized AgNPs changes from straw-yellow to reddish brown within 72 h of incubation at 45°C at 200 rpm in a shaker incubator. The spectra of AgNPs showed maximum absorption at 420 nm, indicating the formation of AgNPs due to the excitation of surface plasmon vibrations in AgNPs.

### 3.6 Electrochemical ORR activity and charge transport properties of biogenic silver

The ORR mechanism is studied using various electrochemical techniques, such as CV. The reduction of O_2_ to OH^−^ occurs on two competing ORR mechanisms. The first process occurs when coupled with oxidation at the anode; this four-electron process rapidly combines O_2_ with charged particles to generate water as the end product. The second method, which is less efficient, entails two phases, one of which uses the intermediary H_2_O_2_ (Eq. 2) and either reduces two electrons in H_2_O_2_ or increases with the reaction medium (Eq. 3b). AgNPs favor ORR pathways with four electrons and suppress the production of harmful peroxide.

$$O_{2}+4H+4e^{-}\to \;2H_{2}O$$ (1)

$$O_{2}+2H_{2}O+2e^{-}\to \;{\text{HO}}_{2}^{-}+{\text{OH}}^{-}$$ (2)

$${\text{HO}}_{2}^{-}+H_{2}O+2e^{-}\to \;3{\text{OH}}^{-}$$ (3a)

$$2{\text{HO}}_{2}^{-}\to \;4{\text{OH}}^{-}+O_{2}$$ (3b)

**FIGURE 7:**
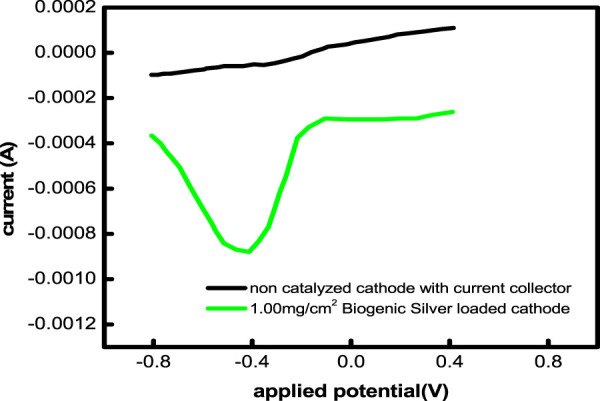
Linear sweep voltammograms of a noncatalyzed cathode and a 1 mg/cm^2^ AgNP-loaded cathode in an air-saturated 1 M KCl solution.

The electrochemically produced Ag activity was studied by using CV in an air-saturated environment. In a solution of 1.0 M KCl, all forms of synthetic and biogenic AgNP catalysis showed a distinctive oxygen reduction peak at approximately 0.5 V. This oxygen reduction peak is brought on by the proton insertion onto biogenic silver. For the synthetic biogenic AgNPs, the exact peaks of oxygen reduction are located at 0.43, 0.42, 0.410, and 0.39 V, respectively (Figure 7). The fall from high capacity, which increases the ORR activity of the associated catalyst, causes the oxygen reduction highpoint to change into a less negative potential. Furthermore, 1.58, 4.6, and 7.34 times higher current is needed to reduce biogenic AgNPs. Greater current flows via the biogenic AgNPs, which have a larger energetic surface area, less diffusion resistance to positively charged particles, and easier electrolyte penetration. A nanoparticle works well as a catalyst in the current work because of these factors. Between the highest biogenic AgNP (0.70 V) and the lowest biogenic AgNP, the separations reveal the maximum anode and cathode peak (0.616 V). This demonstrates the alteration in the electrode component’s recovery. Lesser peak separation indicates decreased non-reversible biogenic AgNP (0.687 V) electrodes.

EIS analysis is frequently used to determine the charge transport properties of electroactive components within the electrode/electrolyte association (Magar et al., 2021). A Nyquist plot (imaginary component vs. actual component of impedance) is used to observe the associated electrochemical property of the electrode (Ruhi et al., 2014). In the maximum frequency range, each point has a distinct semicircle, while in the minimum frequency range, it has a straight line. The diameters are used to calculate the charge transfer resistance (Rct) of each electrode (Mamlouk, 2021). The electrolyte and the Rct values have a directly proportional relationship in the associated reaction between the catalyst and the reactant. At a synthetic biogenic AgNP electrode (35.05+), the estimated value of Rct is seen to follow that of a naked paper-with-carbon electrode (102.30+). The biogenic AgNP electrode exhibits substantial mobility while having a lower Rct value. The electron movement should be faster to enhance the rate of reduction that corresponds to the maximal reduction current received from the biogenic AgNPs (He et al., 2019). The decreased Rct is the cause of the increasing ORR over the potential for biogenic AgNPs. Compared to carbon particles and single-dimensional nanoparticles, biogenic AgNPs exhibit the highest ORR, with good charge movement activity (Patil and Chandrasekaran, 2020). This is because their two-dimensional structure makes the best support matrix for biogenic AgNPs and promotes maximal connection. Due to their maximal adsorption properties, biogenic AgNPs are regarded as an ideal option for the adsorbent component and the catalyst (Estevez et al., 2020). Additionally, the high electronic conductivity of biogenic AgNPs with the 2D planar conjugation structure may be used to effectively transport an electron to the biogenic AgNPs where the oxygen is reduced electrochemically with the amplification of the electrochemical achievement.

### 3.7 Power generation from MFCs with biogenic AgNPs

**FIGURE 8:**
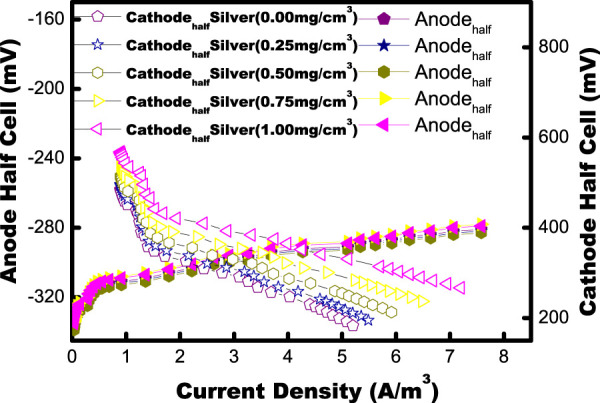
Polarization plots with different MFC: Biogenic AgNP-free and different concentrations of biogenic AgNPs on the cathode (half-cell). The data points for the anode and cathode half-cell potentials are represented by solid and open symbols on the graph.

Each group of MFC test cycles is processed for 34 h (±3 h). At first, the anaerobic anode of the MFC chamber is mixed with synthetically made acetate wastewater without inoculation for approximately a day (24 h) before feeding. A neutral pH (pH) of 7.0 ± 0.2 is maintained for the wastewater. A capacity of 197 ± 9 mV in the anodic half-cell is observed, which is in accordance with the Ag/AgCl reference electrodes with no power supply. This implies the nonpresence of the biotic process in the chamber in the absence of inoculum. Anaerobic consortia gathered from below the sludge of the septic tank are used to inoculate the anodic chamber. MFCs are maintained in an open-circuit mode until they become adapted to the environment. Then, they are operated in a closed-circuit configuration in fed-batch mode at room temperature. After inoculation, the capacity of the anodic half-cell starts falling to donate the electrons by anodephile to the anode side, and, at the plateau, it gained a value of approximately −282 ± 8 mV opposite to an external resistance on every MFC of 90–10Ω. Stable performance was gained in the anode half-cell after five cycles (Figure 8).

Stable anode and cathode half-cells with various concentrations of AgNPs were calculated during the current work (Jehnichen et al., 2019). The effect of different concentrations of AgNPs on the generation of power was observed by testing 0, 0.25, 050, 0.75, and 1.0 mg/cm^2^ loads of AgNPs. The wide difference in the potential of the cathodic half-cell is listed with various loads of biogenic AgNPs in the air cathode of the MFC. The cathode without biogenic AgNPs (catalyst-free) produces the highest possible volumetric power density (PD_max_) is 0.59 W/m^3^. With a 0.25, 0.50, 0.75, and 1.0 mg/cm^2^ load of biogenic AgNPs, the PD_max_ of the MFC is raised to 1.5, 2.5, 3.97 and 4.70 W/m^3^. Increasing the amount of AgNPs from 0 to 0.25 mg/cm^2^ almost doubled the PD_max_. The increase from 0.25 to 1.0 mg/cm^2^ AgNPs improved PD_max_ by 26.30%. As the biogenic AgNPs load on the cathode increased, several key observations were made: the highest open-circuit potential, CE and the efficiency of chemical oxygen demand (COD) removal both demonstrated an upward trend, while the internal resistance notably decreased. It can be concluded that the biogenic AgNP electrode affects the generation of power in MFCs. The decrease in internal resistance with biogenic AgNPs contributed to the high reduction of O_2_ kinetics at the surface of the cathode.

The MFCs achieved constant maxima in their open-circuit potential (OCP) polarization studies. At the point when the OCV was steady, an electrochemical examination of the MFC was carried out using a multimeter and data-collecting device to measure polarization and power density curves throughout a range of resistance values (90 kΩ–10Ω) as shown in Figure 8. The half-cell potential showed the difference in the cathode half-cell, while the anode half-cell magnitude did not vary with various external resistances. The results clearly suggested the effect of AgNPs as cathode catalysts on oxygen reduction. Compared to the catalyst-free cathode (OCP 625 mV and *PD*_max_ ∼0.59 W/m^3^), the existence of Ag in the cathode promotes greater OCP (814 mV) and higher *PD*_max_ (4.70 W/m^3^), as shown in Table 1.

**TABLE 1:** Effect of the biogenic silver-based air cathode in the MFCs compared with the benchmark Pt/C. For comparison, a fixed quantity of the catalyst (0.50–1.00 gm/cm^2^ biogenic silver) was loaded to the carbon support.

MFCs with different cathodes	Catalyst-free	Biogenic silver (0.50 mg/cm^2^)	Biogenic silver (0.75 mg/cm^2^)	Biogenic silver (1.00 mg/cm^2^)	Benchmark Pt/C
Maximum OCP (mV)	625	750	800	814	840
Maximum volumetric power density (W/m^3^)	0.59	2.5	3.97	4.70	5.69
Maximum CE (%)	5.5	8.5	11.4	11.7	13.0
COD removal efficiency (%)	69.3	78.8	82.5	83.8	84.40
Internal resistance (Ω)	175	109	99	87	77

**FIGURE 9:**
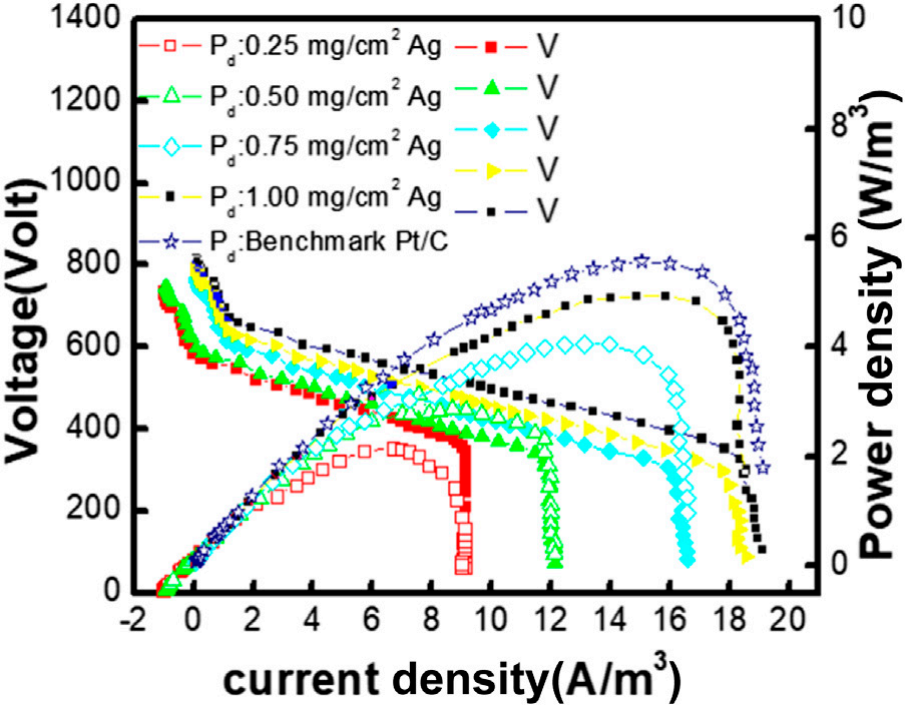
Polarization plots with different MFCs: biogenic AgNP-free and different concentrations of biogenic AgNPs on the cathode. The data points for power density and voltage are represented by solid and open symbols on the graph.

When the external resistance increases, the power generation decreases, which is a typical characteristic of a fuel cell. A very rapid potential drop is observed at minimum external resistance for MFCs with cathodes without catalyst. When biogenic AgNPs were used, power generation was enhanced, which can be clearly observed as the *PD*_max_ without and with the biogenic AgNPs were 3.96 W/m^3^ and 4.69 W/m^3^, respectively. It shows the good conductivity of the biogenic silver (Figure 9). The current- interruption method was used to estimate the internal resistance, and it was measured to be 175, 109, 99, 87, and 77 Ω with catalyst-free biogenic AgNPs and Pt/C cathode catalysts, respectively. The PD_max_ of the MFC with the standard Pt/C cathode was 5.69 W/m^3^, which is 21.15% greater than that of the biogenic AgNP cathode. Due to the maximum performance-to-cost ratio (discussed in the following paragraphs), the Pt/C cathode could be replaced with a biogenic AgNP cathode based on this result. Biogenic AgNPs become the advisable candidates compared to the different forms of carbon due to their maximum surface area, good conduction of electricity, and lower production cost. Greater power generation capability is shown by the biogenic AgNP cathode than an chemically produced silver cathode (0.775 W/m^3^), a hydrothermally formed biogenic silver (2.54 W/m^3^), and a hydrothermal produced biogenic AgNP cathode (8.3 ± 0.4 mg/cm^2^ loading) with conductive support made up of graphite (0. 468 ± 0.020 W/m^3^). The combined reaction of the *α*-biogenic silver phase, biogenic AgNP morphology, greater crystallinity, and the primary electrochemical characteristic (ORR performance and greatest charge transport) causes the higher PD_*max*_ observed in the current project.

The generation of power is controlled by the potential of the cathode cover in MFCs. It is seen that a higher driving force with an overcharge of 0.32 ± 0.015 is needed for the cathode to compare the anode value required (0.6 ± 0.06 V). If a cathode is catalyst-free, the fast decline in cathode potential from OCP gives a smaller kinetic reaction. The density of the cathode half-cell was observed in the following order: Pt/C > Ag nanoparticle > catalyst-free electrode at each resistance value. This series shows the catalytic activity order. The comparison of the current density from biogenic AgNPs with that of the Pt/cathode shows the higher ORR activity in biogenic AgNPs and higher electrical characteristics of biogenic AgNPs.

The measured cathodic half-cell potential of the Pt/C and Ag-based electrodes exhibits a gradual increase over time, which deviates from the conventional trend observed in many cathodes where the potential tends to decrease with the passage of time. This distinctive phenomenon underscores the efficacy of catalysts based on biogenic silver nanoparticles (AgNPs), which demonstrate comparable behavior to catalysts based on platinum/carbon (Pt/C). With a cation exchange membrane (CEM), the catholyte pH increases due to the rise of the cathodic half-cell potential because other cations (such as Na^+^ and K^+^) are migrated apart from the positively charged particles in the catholyte. The enhanced ORR takes place in a basic (alkaline) environment due to the presence of biogenic AgNPs. As a result of the concentration gradient, all cation species move from the anode to the cathode, leading to an observable pH difference in the Microbial Fuel Cell (MFC) environment. Because of this, pH inequality is a noticeable occurrence in the MFC situation. Due to the formation of OH by electrolysis of water (Eqs 1–3b) at the cathode area, along with ORR, mainly within the nonbuffered surroundings in the systems with a membrane, the pH may increase.

Although alkalinity adversely affects the performance of several cathodes in nonbuffered environments, the opposite trend in the present work is attributed to the role of biogenic AgNPs, which facilitate ORR because the alkalinity seems to have a significant effect on the performance of many cathodes. Many research associations revealed that biogenic AgNPs show excellent catalytic characteristics for oxygen reduction in an alkaline medium. Oxygen reduction on a biogenic AgNP electrode in a basic medium goes through a four-electron pathway instead of a two-electron pathway.

## 4 Cost estimation of cathode catalysts

The estimated value for a biogenic AgNP cathode depends on the retail cost of the substances used in manufacture. The commercial value for a cathode and a Pt load is as simple as the retail cost value. The main costs of manufacturing pure biogenic AgNPs that contain approximately 80% biogenic AgNP loading, including consumption of electricity is $ 3.81 per gram. The cost of a Pt/cathode with 20% Pt loading is $30 per gram. Because the cost of a Pt/cathode catalyst is 60 times more than that of a biogenic AgNP with equivalent loads, using biogenic AgNPs as ORR catalysts instead of expensive Pt in MFC cathodes could be highly beneficial. Biogenic AgNPs have huge potential for various commercial uses in MFCs.

## 5 Wastewater treatment

The MFC is a viable wastewater treatment system that generates electricity (Tsekouras et al., 2022). The efficiency of MFCs shows that chemical oxygen demand (COD) can be removed from wastewater (Munoz-Cupa et al., 2021). The beneficial function of mixed microflora in wastewater treatment is demonstrated by high COD elimination. COD removal in MFCs rose as biogenic AgNP concentration increased, with the greatest stable COD removal of 78.8% achieved with an AgNP concentration of 0.50 mg/cm^2^. The increased concentration of biogenic AgNPs demonstrated a further increase in the COD removal capacity to 78.8% and 83.8%, respectively, which is close to the maximum COD removal ability of 84.40% (Table 1). Because CE is directly related to wastewater treatment, the highest CE obtained from MFCs with oxygen-deficient biogenic AgNP cathodes (12.1%) was also near that of the standard Pt/C cathode (12.6%), implying that biogenic AgNPs could play a significant role in the subsequent reduction reaction and improve wastewater treatment kinetics. The higher rate of electron and proton consumption increases COD elimination and energy output in a microbial electrochemical system.

## 6 Biofilm formation analysis on a biogenic AgNP electrode

The development of biofilm and its constituent parts on the different concentrations of biogenic AgNP electrode surfaces was studied using CLSM. Figure 9 describes the quantifiable outcomes of biofilm formation on various biogenic AgNP electrodes during MFC studies.

**FIGURE 10:**
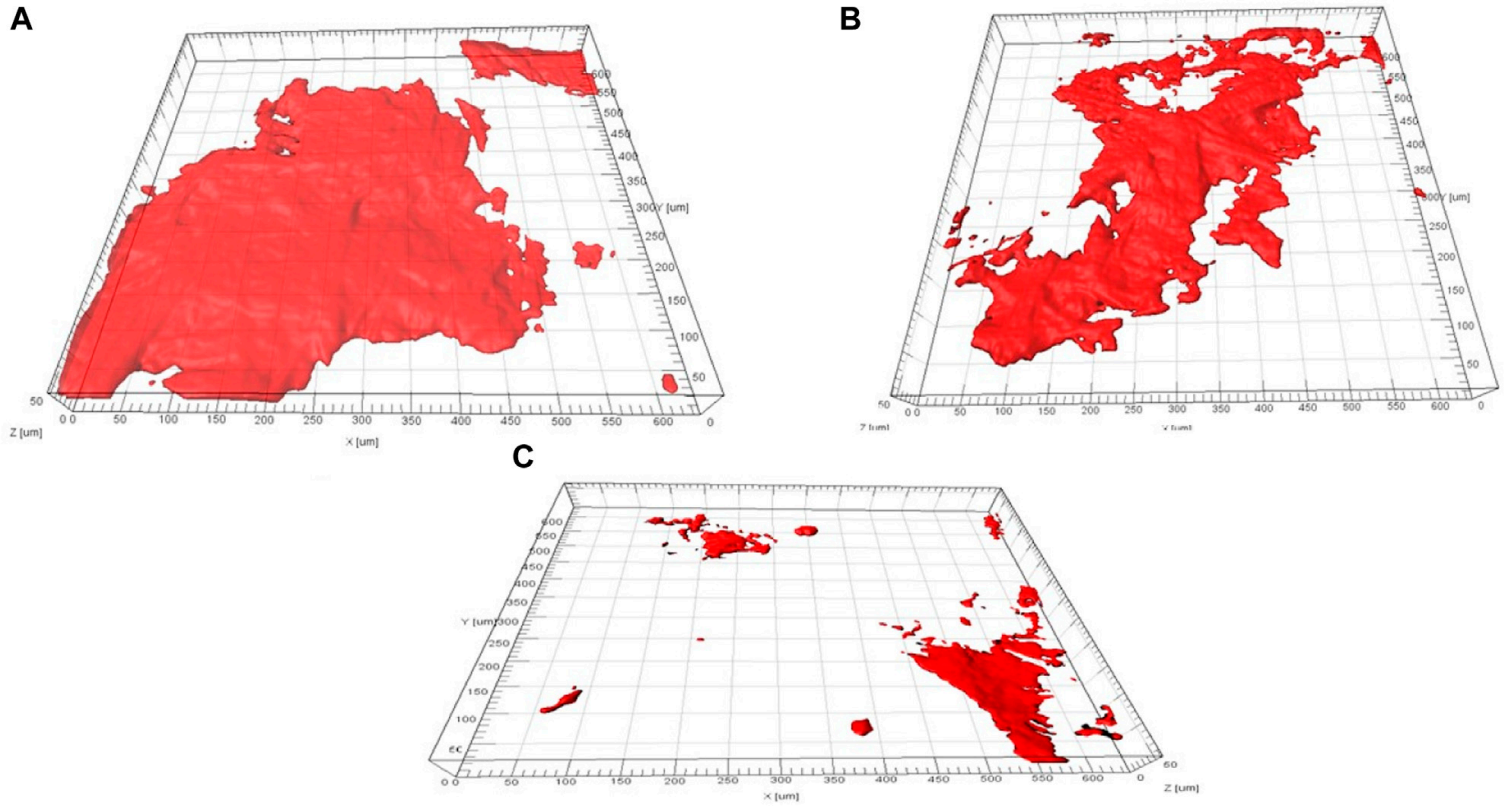
Biofilm formation analysis of a biogenic AgNP electrode. **(A)** 0.0 mg/cm^2^ loading of the biogenic AgNP electrode, **(B)** 0.25 mg/cm^2^ loading of the biogenic AgNP electrode, and **(C)** 1.0 mg/cm^2^ loading of the biogenic AgNP electrode.

The data in Figure 10 were computed using the COMSTAT program. A notably higher biomass of cells was observed in the 0.0 and 0.25 mg/cm^2^ biogenic AgNP electrodes. However, less biomass growth was observed in the 1.0 mg/cm^2^ biogenic AgNP electrode. The minimum and maximum biovolumes of the cell layers for the 0.0, 0.25, and 1 mg/cm^2^ biogenic AgNP electrodes were measured as 14.78 m^3^/m^2^ (1.72), 12.59 m^3^/m^2^ (1.76), and 5.47 m^3^/m^2^ (0.61), respectively. The biovolumes of the cells decreased with increased concentration. The presence of biogenic AgNPs resulted in increased inhibition of biofilm growth, as seen in Figure 10. Increased growth of biofilm was observed in the absence of biogenic AgNPs. This demonstrates the antimicrobial effect of biogenic AgNPs. The Imaris 3D pictures confirm the findings of the COMSTAT analysis. From the Imaris picture, it can be seen that the 0.0 mg/cm^2^ biogenic AgNP cathode had a substantial amount of living cells present; in contrast, the 1.0 mg/cm^2^ biogenic AgNP cathode had less EPS and less apparent live cells (Figure 10).

## 7 Conclusion

This study describes an effective approach for the hydrothermal production of biogenic AgNPs and their use in MFC cathodes, which demonstrated performance comparable to Pt catalysts for the ORR. The study reveals that the amount of biogenic AgNPs on graphene sheets has a direct correlation with the operational efficiency of MFCs, taking into account parameters such as the open-circuit potential (OCP), maximum power density (PD_max_), CE, COD removal efficacy, and decreased electrical resistance. Graphene appears to be the most effective carbon support material tested, with optimum structural and electrical properties. Surprisingly, MFCs using biogenic AgNPs outperform those using the traditional Pt/crystal catalyst in terms of overall performance. Notably, cathode catalysts based on AgNPs improve the alkalinity of MFC cathodes to the same extent as the Pt/C catalyst. The key finding is that these results indicate that biogenic AgNPs have the potential to replace Pt as the preferred cathode catalyst in MFC applications, particularly in large-scale wastewater treatment plants. This study provides a detailed explanation of the possibility of incorporating biogenic AgNPs into MFCs, ushering in a promising era for enhanced wastewater treatment and sustainable energy generation.

## Data Availability

The original contributions presented in the study are included in the article/Supplementary Material; further inquiries can be directed to the corresponding authors.
